# The Role of Dark and Vulnerable Personality Traits in Self-Harm, Suicide, and Risky Behaviors Among Young Adults in Tehran

**DOI:** 10.1192/j.eurpsy.2025.775

**Published:** 2025-08-26

**Authors:** K. Qaderi Bagajan

**Affiliations:** 1 Department of Clinical and Health Psychology, Shahid Beheshti University, Tehran, Iran, Islamic Republic Of; 2 Psychological Diagnostics group, Department of Psychology, Humboldt-Universität zu Berlin, Berlin, Germany

## Abstract

**Introduction:**

Suicidal behaviors, non-suicidal self-injury, and risky behaviors are significant concerns in young adulthood.

**Objectives:**

This study investigates the causal relationships between dark and vulnerable personality traits, guilt and shame proneness, and these behaviors among young adults (ages 18-40) in Tehran. Adopting a dimensional and dynamic perspective on personality, the study aims to develop a comprehensive model that incorporates proposed pathological personality traits from DSM-5 (PID-5).

**Methods:**

Using a cross-sectional design and structural equation modeling, the study analyzed data from a large sample of 1,876 participants, including both a general population sample (n=1,696) and a clinical population (n=180) with suicidal/self-harm behaviors or personality disorders. Data were collected using the Short Dark Tetrad (SD4), Triple Scales of Vulnerable Dark Personality Traits, Guilt and Shame Proneness Scale (GASP), Domain-Specific Risk-Taking (DOSPERT), Self-Harm Inventory (SHI), and Suicidal Behaviors Questionnaire-Revised (SBQ-R).

**Results:**

The study evaluated three main models and nine sub-models. Findings from the comprehensive research model indicated that the proposed causal model, incorporating dark and vulnerable personality traits and guilt and shame proneness, effectively explains self-harm, suicidal, and risky behaviors in both young adults and those with diagnosed personality disorders. Key findings include:

A spectrum of dark and vulnerable personality traits significantly influences self-harm, suicidal, and risky behaviors. Pathological shame proneness, particularly when accompanied by detachment, increases suicidal tendencies. The absence of healthy guilt and shame proneness is associated with higher levels of risky behaviors and non-suicidal self-injury. All dimensions of the dark and vulnerable personality spectrum predict lower healthy guilt and shame proneness and higher pathological shame proneness.

Finally, Healthy guilt and shame proneness plays a mediating and protective role, reducing the likelihood of self-harm and risky behaviors.

**Conclusions:**

This study contributes to the conceptualization of self-harm, suicidal, and risky behaviors within a dimensional and spectrum-oriented framework, considering personality traits, moral emotions, and behavioral consequences. Practical and research implications are discussed.

**Keywords:**

Dark Personality Traits, Risky Behaviors, Self-Harm Behaviors, Suicide, Guilt and Shame Proneness, Vulnerable Personality Traits, Young Adults.

**Disclosure of Interest:**

None Declared

